# Seasonal and interannual variability of the free‐living and particle‐associated bacteria of a coastal microbiome

**DOI:** 10.1111/1758-2229.13299

**Published:** 2024-07-31

**Authors:** Isabel Ferrera, Adrià Auladell, Vanessa Balagué, Albert Reñé, Esther Garcés, Ramon Massana, Josep M. Gasol

**Affiliations:** ^1^ Department of Marine Biology and Oceanography Institut de Ciències del Mar (ICM‐CSIC) Barcelona Catalonia Spain; ^2^ Centro Oceanográfico de Málaga, Instituto Español de Oceanografía (IEO‐CSIC) Málaga Spain; ^3^ Present address: Institut de Biologia Evolutiva (IBE‐UPF‐CSIC) Barcelona Catalonia Spain

## Abstract

Marine microbial communities differ genetically, metabolically, and ecologically according to their lifestyle, and they may respond differently to environmental changes. In this study, we investigated the seasonal dynamics of bacterial assemblies in the free‐living (FL) and particle‐associated (PA) fractions across a span of 6 years in the Blanes Bay Microbial Observatory in the Northwestern Mediterranean. Both lifestyles showed marked seasonality. The trends in alpha diversity were similar, with lower values in spring–summer than in autumn‐winter. Samples from both fractions were grouped seasonally and the percentage of community variability explained by the measured environmental variables was comparable (32% in FL and 31% in PA). Canonical analyses showed that biotic interactions were determinants of bacterioplankton dynamics and that their relevance varies depending on lifestyles. Time‐decay curves confirmed a high degree of predictability in both fractions. Yet, ‘seasonal’ Amplicon Sequence Variants (ASVs) (as defined by Lomb Scargle time series analysis) in the PA communities represented 46% of the total relative abundance while these accounted for 30% in the FL fraction. These results demonstrate that bacteria inhabiting both fractions exhibit marked seasonality, highlighting the importance of accounting for both lifestyles to fully comprehend the dynamics of marine prokaryotic communities.

## Graphical abstract

Differentiating marine microbial communities by their lifestyle is essential to understand their responses to environmental changes since they differ genetically, metabolically, and ecologically. Bacteria inhabiting the free‐living (FL) and particle‐associated (PA) fractions in the Blanes Bay Microbial Observatory (NW Mediterranean) exhibit marked seasonality.

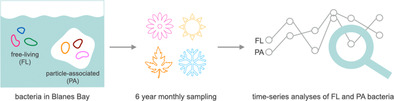

## INTRODUCTION

Microbial communities change temporally at different time scales, from diel to decadal, and probably beyond. Increasing efforts are being invested towards exploring temporal changes in marine microbes and understanding the controls driving these changes. Microbial observatories provide valuable data that can be used to develop models on how marine microbiomes will behave in future global change scenarios (Bunse & Pinhassi, 2017; Giovannoni & Vergin, 2012). By studying time series data we can detect periodic patterns or identify unexpected variability that make microbial community behaviour unpredictable. The implementation of these microbial observatories in oceanic and coastal monitoring stations has taken place worldwide (see reviews by Bunse & Pinhassi, 2017; Buttigieg et al., 2018) and, although long‐term studies are mostly from mid‐latitudes, they have revealed that deterministic mechanisms play important roles in assembling communities and that bacterioplankton exhibit repeatable and predictable patterns over extended periods (i.e., Alonso‐Sáez et al., 2015; Cram et al., 2015; Eiler et al., 2011; Fuhrman et al., 2015; Gilbert et al., 2012; Lambert et al., 2019). In temperate regions, prokaryotic communities are primarily governed by seasonal parameters such as day length, temperature, nutrients, and chlorophyll *a* concentration (i.e., Andersson et al., 2010; Fuhrman et al., 2015; Gilbert et al., 2009; Lambert et al., 2019; Pinhassi et al., 2006; Sapp et al., 2007). In addition to environmental variability, biological interactions could have a more important role in controlling plankton dynamics than previously thought (Needham & Fuhrman, 2016; Yeh & Fuhrman,  2022b).

Most of these studies have focused on the free‐living (FL) fraction of bacterioplankton or in the bulk community, while less effort has been put into exploring the seasonality of particle‐associated (PA) communities. Although size‐fractionation represents an additional cost and time effort, differentiating marine microbial communities by their lifestyle is essential to understanding their responses to environmental change since they differ genetically, metabolically, and ecologically (Crespo et al., 2013; Grossart, 2010; Mestre, Borrull et al., 2017; Mestre, Ferrera et al., 2017; Rieck et al., 2015; Salazar et al., 2015; Shen et al., 2022; Yu et al., 2023). The scarce examination of microbial communities in different size fractions at a seasonal scale has reported substantial differences among them. For example, a study conducted at the Pivers Island Coastal Observatory (North Carolina, USA) over a year found that particle‐associated communities, particularly those associated with larger particles (>63 μm), were more variable over time than the free‐living ones (Yung et al., 2016). Particle‐associated communities were less responsive to the commonly measured variables, such as temperature, chlorophyll concentration or nutrients, suggesting that other factors drive these communities. A report from open waters of the Eastern Mediterranean Sea that sampled 6 times over 2 years reached similar conclusions; none of the season‐related measured parameters could be linked to the variability of particle‐associated communities (>11 μm) (Roth Rosenberg et al., 2021). Associations with other organisms and/or differences in particle composition were hypothesized as potential drivers of particle‐associated communities in both studies (Roth Rosenberg et al., 2021; Yung et al., 2016). Additionally, a comparison of bacterial communities along the particulate matter continuum (6 fractions ranging from 0.2 to 200 μm) over 2 years in the Blanes Bay Microbial Observatory (NW Mediterranean) found that community changes over time were stronger towards the larger size fractions (Mestre et al., 2020). As opposed to the abovementioned reports, the later study reported that sea surface temperature and day length were overall good predictors of community changes and that each fraction was additionally driven by a particular combination of other environmental factors. Although these studies offer a valuable vision of prokaryotic dynamics at the short‐term scale (1–2 years), longer observations are needed to assess the temporal stability of these patterns. In that sense, Yeh and Fuhrman (2022a) recently characterized microbes from two size fractions (0.2–1 and 1–80 μm) at the San‐Pedro Ocean Time series over 14 years and found that, at this site, free‐living and particle‐associated prokaryotic communities were stable over time. Moreover, this study reported that biotic interactions determine to a large extent the temporal dynamics of prokaryotes, with varying importance of specific interactions depending on their lifestyle.

The stability ‐or lack of stability‐ of particle‐associated prokaryotic communities may depend on the nature and periodicity of particle formation through biotic and abiotic processes. Particles are heterogeneous in composition (living cells, detrital particles, abiotic particles, etc.) and origin (generated in situ by biological production mostly by phytoplankton and zooplankton (Guidi et al., 2009; Kiørboe & Hansen, 1993; Ploug et al., 2008), or from allochthonous sources, such as sediment resuspension, aerosol deposition or terrestrial runoff). Seasonal variations in phytoplankton and zooplankton could influence the production of particles of biological origin, while particles of abiotic origin would be more susceptible to environmental perturbations, such as extreme weather events.

In this paper, we investigate the seasonal and interannual variability of free‐living and particle‐associated bacterial assemblies over 6 years in the Blanes Bay Microbial Observatory, a coastal station with limited terrestrial influence in the NW Mediterranean Sea. Using high‐throughput sequencing of the 16S rRNA gene, we compare for the first time the diversity and stability patterns between the two fractions in this oligotrophic site and determine whether they are equally influenced by environmental seasonality and/or by biotic interactions in the long term. Building on previous research by Mestre et al. (2020) and given that Blanes Bay is subjected to strong predictable fluctuations in environmental conditions with minimal stochastic perturbations, and that pelagic small particles are largely produced by seasonally changing phytoplankton and zooplankton (Calbet et al., 2001; Nunes et al., 2018), we hypothesize that both fractions will present a marked seasonality. Considering that particle production may exhibit its seasonality, the patterns of seasonality may however not necessarily mirror each other.

## EXPERIMENTAL PROCEDURES

### Location and sample collection

Samples were collected in the Blanes Bay Microbial Observatory, a station located in the NW Mediterranean about 1 km offshore (41°40′ N, 2°48′ E) over a water column of 20 m depth. Sampling was conducted monthly over 6 years, from January 2004 to December 2009. Water temperature and salinity were measured in situ with a conductivity, temperature, and depth probe, and light penetration was estimated by using a Secchi disk. Surface seawater was pre‐filtered through a 200‐μm nylon mesh, transported to the laboratory under dim light in 20‐L plastic carboys, and processed within 2 h. Chlorophyll *a* concentration was obtained by fluorometry from acetone extracts of samples collected on GF/F filters (Yentsch & Menzel, 1963). The concentration of inorganic nutrients (NO_3_^−^, NO_2_^−^, NH_4_^+^, PO_4_^3−^, SiO_2_) was determined spectrophotometrically using an Alliance Evolution II autoanalyser (Grasshoff et al., 1983). Bacterial biomass production was estimated as described in Alonso‐Sáez et al. (2008). The abundance of picocyanobacteria, high and low nucleic acid populations of heterotrophic prokaryotes, and phototrophic pico‐ and nanoeukaryotes was measured by flow cytometry as described elsewhere (Gasol & Moran, 2015). The concentration of phototrophic and heterotrophic flagellates in different size ranges was measured by epifluorescence microscopy in 0.6‐μm black polycarbonate filters (Giner et al., 2016).

Microbial biomass was collected by filtering about 4 L of seawater using a peristaltic pump sequentially through a 20‐μm nylon mesh (to remove large eukaryotes), a 3‐μm pore‐size 47 mm polycarbonate filter and a 0.2‐μm pore‐size Sterivex unit (Millipore). The 3‐μm filter was changed if there was a decline in the flow rate to prevent the retention of free‐living bacteria in the prefilter due to clogging. Sterivex units and the 3‐μm filters were stored at −80°C. The serial filtration allowed the separation of two bacterioplankton fractions: the particle‐associated (PA; 3–20 μm) and the free‐living (FL; 0.2–3 μm) bacteria.

### DNA extraction, PCR amplification and sequencing

DNA was extracted with lysozyme, proteinase K, and sodium dodecyl sulfate, and a standard phenol‐chloroform‐isoamyl alcohol protocol as described in Massana et al. (1997). Extracted DNA was purified and concentrated in an Amicon 100 (Millipore) and quantified in a NanoDrop‐1000 spectrophotometer (Thermo Scientific). DNA was stored at −80°C and an aliquot from each sample was sent for sequencing to the Research and Testing Laboratory (Lubbock, TX, USA; http://rtlgenomics.com/). Primers 341F (5′‐CCTACGGGNGGCWGCAG‐3′) (Herlemann et al., 2011) and 806RB (5′‐GGACTACNVGGGTWTCTAAT‐3′) (Apprill et al., 2015) were used to amplify partial 16S rRNA genes. These primers are optimal for the amplification of Bacteria but do not recover the Archaea domain (McNichol et al., 2021). Sequencing was performed in an Illumina MiSeq sequencer (2× 250 bp).

### Sequence processing

DADA2 v1.4 was used to define the 16S rRNA gene V3‐V4 amplicon sequence variants (ASVs) and remove chimeras (parameters: maxN = 0, maxEE = 2,4, trunclen = 230,225; Callahan et al., 2016). Previously, spurious sequences and primers were trimmed using *cutadapt* v.1.16 (Martin, 2011). Taxonomic assignment of the ASVs was performed within DADA2 with the Ribosomal Database Project Bayes naïve classifier (Cole et al., 2014) against the SILVA database release v138 (Quast et al., 2013). ASVs classified as mitochondria or chloroplast were removed. After data processing, an average of 30,845 sequences per sample were retained (Min: 6645, Max: 143637). A total of 72 samples from the free‐living fraction and 69 from the particle‐attached fraction were included in the analyses.

### Statistical analyses

Data analyses were performed using the R language (v3.5) (R Development Core Team, 2015); data was managed using packages *phyloseq* (v1.26) and *tidyverse* (v1.3), and all visualizations were done using *ggplot2* (v3.2). To compare ecological patterns between rare and abundant taxa, the relative abundance threshold of 1% per sample was used (i.e., rare taxa are those having abundances below 1% of total reads per sample, and abundant taxa are those above this threshold, following Campbell et al., 2011). Among the abundant ASVs, taxa were further classified depending on their occurrence and following the distribution definitions of Chafee et al. (2018); taxa presenting a ‘broad’ distribution were those appearing in ≥75% of the samples, ‘intermediate’ those present in >10% and <75% of samples, and ‘narrow’ those appearing in ≤10% of the samples. To test for lifestyle preference, that is whether an ASV presented a preference for the particle‐associated or the free‐living fraction, the ‘particle‐association niche index’ (PAN index) was used as described in Salazar et al. (2015). To evaluate seasonality, that is, repetitive occurring patterns over time due to changes in environmental conditions, we tested whether ASVs presented a periodic pattern. We used the Lomb Scargle periodogram (LSP) as implemented in the *Lomb* package v1.2 (Ruf, 1999). The Lomb Scargle is a method to estimate the frequency spectrum to find significant repetitive signals. This method can also account for unevenly sampled signals, a typical problem with long‐term analyses (see Lambert et al., 2019 for a detailed explanation). Only ASVs presenting a peak normalized power (pNmax) above 10 were considered to show a seasonal behaviour, using the same criteria as Auladell et al. (2022).

Alpha diversity was analysed using the Chao1 (richness) and Shannon (richness and evenness) diversity indices using a rarefied table to the lowest number of sequences (6645). Community distance was tested using Bray–Curtis dissimilarity after rarefaction and square root transformation. Statistical differences between groups were tested by Permutational MANOVA (PERMANOVA) analysis with the *adonis* function in the *vegan* package (v2.5). Additionally, beta diversity was also computed using the phylogeny‐based weighted UniFrac distances. To test for recurrent patterns of beta diversity, a time‐decay analysis of the assemblage was computed excluding rare ASVs as recommended elsewhere (Faust et al., 2015). A Mantel test was used to evaluate the similarity between each fraction and (a) the phytoplankton community structure, determined through pigment measurements (Nunes et al., 2018), and (b) the pico‐ and nanoplankton protistan community, determined by analysis of the 18S rRNA gene (Giner et al., 2019). Both datasets were previously standardized prior to these analyses through Hellinger transformation for community data and scaling to the mean for the pigment data. Additionally, a partial canonical constrained analysis (pCCA) was performed following Yeh and Fuhrman (2022b) to determine whether the mantel test analysis was robust to the removal of the effect of environmental variables.

## RESULTS

### Environmental variability

Surface water temperature ranged from minimal mean values in February (12.6°C) to maximal values in August (24.5°C) showing a seasonal trend (Figure S1) displaced 55 days from maximum values of day length in June (range 9.15–15.18 h). The average salinity was 37.71, oscillating from 35.07 (Oct 2005) to 39.09 (Dec 2006), with relatively lower values at the end of spring and in autumn. Inorganic nutrient concentrations were lower in summer and water transparency measured by Secchi depth was generally higher during that season. Chlorophyll *a* concentrations followed a clear seasonal cycle, with higher values during the winter–spring transition and lowest in summer (ANOVA, *p* < 0.001) (Figure S2). Slightly higher concentrations and activity of heterotrophic bacteria occurred during spring and summer. *Synechococcus* was more abundant in late summer and early autumn, and *Prochlorococcus* in winter. Seasonal trends of each measured variable are depicted in Figures S1 and S2.

### Alpha and beta diversity patterns

**FIGURE 1:**
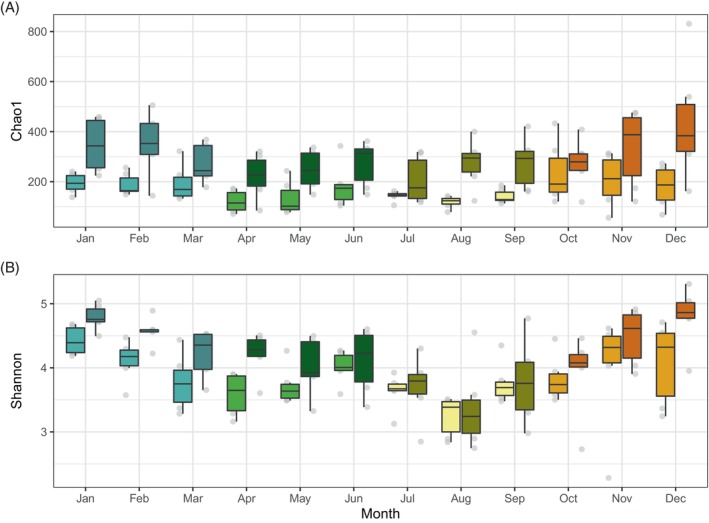
Chao1 index of richness (A) and Shannon (B) diversity distribution of bacterioplankton communities coloured by season and fraction (lighter colours correspond to the free‐living fraction and darker tones to the particle‐associated one). Each boxplot presents the median and interquartile range of the distribution of 6 data points. Whiskers represent 1.5 times the interquartile range.

We examined the patterns of diversity of the free‐living (FL, 0.2–3 μm) and particle‐associated (PA, 3–20 μm) bacterial communities in Blanes Bay. The PA fraction consistently exhibited higher alpha diversity values throughout the year (Wilcoxon Rank test, *p* < 0.0001 for Chao1 and Shannon diversity indices) (Figure 1). The seasonal trends of both fractions closely mirrored each other, with statistically significant lower values occurring in the spring–summer compared to autumn‐winter (Wilcoxon Rank test, *p* < 0.0001 for Chao1 and Shannon in the FL, and *p* < 0.001 for Chao1 and *p* < 0.0001 for Shannon in the PA fraction).

As for community differences (beta diversity), non‐metric multidimensional scaling (nMDS) showed a distinct separation of the samples based mostly on the season (Figure S3), which was a more informative factor than fraction to explain the variability in the data (PERMANOVA, season *R* ^2^ = 0.22, fraction *R* ^2^ = 0.06, *p* < 0.001). We then classified the ASVs as either abundant or rare taxa based on their relative abundance (see Experimental Procedures) and we computed community differences separately. The primary clustering by season and then by fraction was maintained, although both factors had less explanatory weight for the rare compared to the abundant assemblies (Abundant: season *R* ^2^ = 0.23, fraction *R* ^2^ = 0.07; Rare: season *R* ^2^ = 0.14 and fraction *R* ^2^ = 0.02) (Figure S3). Further, to examine the patterns by the two abundance categories, we compared the Bray‐Curtis distances between the FL and PA fraction at each sampling time and found that communities were more similar based on the abundant taxa than on the rare components (Figure S4).

**FIGURE 2:**
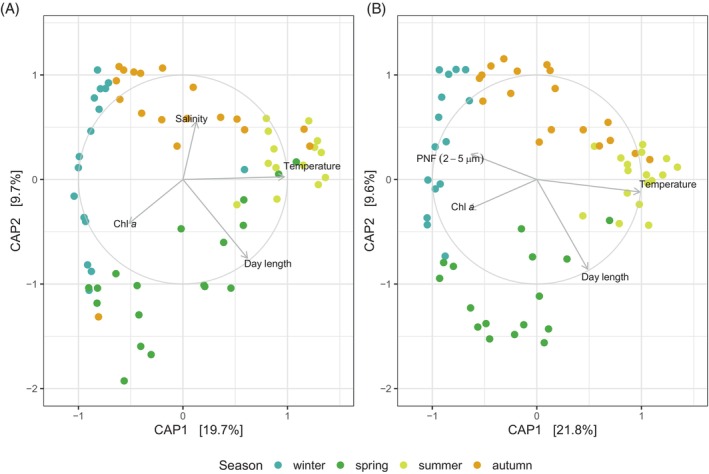
Distance‐based redundancy analysis (dbRD) between community composition at the ASV level and environmental data in (A) the free‐living and (B) the particle‐associated fractions. Explanatory variables are shown in arrows and samples are colour‐coded by season. Analyses are based on the relative abundance of all ASVs (rare and abundant). Chla total: Chlorophyll *a* concentration; PNF 2_5um_Micro: phototrophic nanoflagellates in the 2–5 μm size range counted by epifluorescence microscopy.

Additionally, we investigated the influence of the biotic and abiotic variables in structuring bacterioplankton communities in each size fraction. For that purpose, we used distance‐based Redundancy Analyses (dbRDA) to ordinate samples while constraining the ordination by a set of environmental variables. The results show that FL and PA bacterial communities were separated by season and that the percentage of community variability explained by the first two axes was comparable (29.7% in FL and 31.4% in PA) (Figure 2). Temperature, day length, and chlorophyll *a* concentration were explanatory variables for both bacterial fractions. Besides, salinity (4.1%) contributed to explaining the ordination of free‐living communities whereas for the particle‐associated fraction, the abundance of phototrophic nanoflagellates in the size range of 2–5 μm appeared as a constraint (2.9%).

Further, to investigate if the temporal variation in bacterial communities could be attributed to other microbial components, we performed Mantel tests between the community bacterioplankton matrices of bacteria in each fraction with the matrices of eukaryotic pico‐ and nanoplankton communities based on sequencing of the 18S rRNA gene (data from Giner et al., 2019) and of phytoplankton communities derived from a chemotaxonomic approach based on pigment analysis (data from Nunes et al., 2018). The 18S rRNA gene dataset includes the same fractions as this study, while the phytoplankton chemotaxonomic dataset is based on the analyses of bulk samples, covering the whole phytoplankton size spectrum. All pairs of community composition matrices based on rRNA gene sequencing (that is bacterial vs. protist diversity matrices) showed a good correlation coefficient. For phytoplankton communities, however, the correlation coefficient was higher for PA than for FL communities (Table S1). These correlations may be due in part to seasonal reoccurring patterns in abiotic and biotic variables, such as temperature or chlorophyll *a*, and to the shared environmental preference of different microbial communities. Thus, to validate these results, we used partial canonical correspondence analysis (pCCA) to remove the effects of these two ‘seasonal’ variables (Table S2). The analyses showed that pico‐ and nanoeukaryotic diversity explained temporal variation in both the FL and PA bacteria, but phytoplankton communities only explained variation in particle‐associated bacterial communities.

### Community composition

**FIGURE 3:**
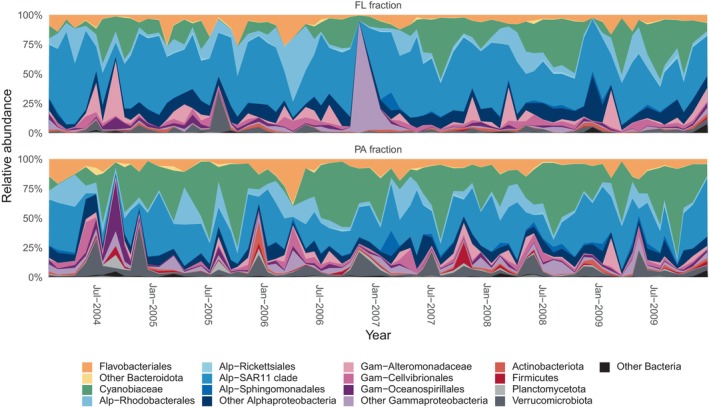
Stacked area plots of the ASVs grouped into the main taxa for the 6‐year time series for the free‐living (upper panel) and the particle‐associated (lower panel) fraction.

We examined the contribution of different broad taxa to the FL and PA bacterial communities (listed in Table S3) and found that the most abundant taxa were fairly similar in both factions (Figure 3). The FL fraction was mainly dominated by the SAR11 clade (median 43.3%), followed by *Cyanobiaceae* (10.4%), Rhodobacterales (5.7%), Flavobacteriales (5%), and Cellvibrionales (2.3%). Similarly, the prevalent taxa in the particulate fraction were the SAR11 clade (24.9%), *Cyanobiaceae*, (23%), Flavobacteriales (6.7), Rhodobacterales (4.6%) and Oceanospirillales (2.8%). Other taxa presented lower relative abundances; among these, the Verrucomicrobia presented higher percentages in the particle‐associated fraction. Note that Archaea were not considered in our study but they are typically present in low abundance in surface waters of Blanes Bay (Alonso‐Sáez et al., 2007).

Regarding the seasonal cycle, the relative contributions of the main groups through the year in both the free‐living and particle‐associated fractions are shown in Figure S5. In the free‐living fraction, the monthly contribution of the SAR11 clade, Rhodobacterales, Alteromonadales, Flavobacteriales and Cyanobiaceae changed considerably. Besides these groups, Verrucomicrobia also varied largely in the particle‐associated fraction. Moreover, the monthly peaks in the relative contribution of each taxonomic group to each fraction seldom occurred simultaneously. For example, the SAR11 clade showed a maximum contribution to the FL communities in May while it was in April for the PA fraction; the *Synechococcus* highest contribution to the FL fraction was in August and to the PA in September. Moreover, even if the mean relative abundance of each broad taxa was similar in the two fractions, a comparison at the ASV level, revealed that the abundances of ASVs shared between fractions (~30% of all ASVs) generally did not show strong correlations, as illustrated in Figure S6 for the top abundant shared ASVs, suggesting that seasonal changes in the relative abundance of these ASVs in either fraction were not synchronous.

### Abundance and occurrence of ASVs

ASVs were classified according to their relative abundance and occurrence (see Table 1). Taxa presenting a ‘broad’ distribution were those appearing in ≥75% of the samples, ‘intermediate’ those present in >10% and <75% of samples, and ‘narrow’ those appearing in ≤10% of the samples. The number of abundant ASVs was similar between the FL and PA datasets (192 and 230 ASVs, respectively). However, the PA fraction contained a greater number of rare ASVs (2265 vs. 4081 for FL and PA), most of which fall into the ‘narrow’ category based on their occurrence. Despite the larger number of rare ASVs in the PA fraction, rare taxa contributed similarly to both communities, accounting for ~17 and 22% of the community structure in FL and PA, respectively.

**TABLE 1:** Number of ASVs (# ASVs), median occurrence, mean abundance (%), and number of seasonal ASVs (# Seasonal ASVs, based on the *lomb scargle* test) for the different categories of ASVs depending on their abundance and occurrence and for each size fraction.

			#ASVs	Median ocurrence	Mean abundance (%)	#Seasonal ASVs
Abundant	FL	Broad	21	86.1	49.4	2
		Intermediate	115	36.1	27.9	43
		Narrow	56	4.2	6.0	0
	PA	Broad	27	89.9	40.8	6
		Intermediate	163	33.3	35.5	79
		Narrow	40	4.3	1.4	0
Rare	FL	Broad	5	81.9	1.1	0
		Intermediate	257	18.1	10.4	47
		Narrow	2003	18.1	5.3	0
	PA	Broad	6	79	1	0
		Intermediate	601	17.4	15.8	131
		Narrow	3474	1.4	5.6	0

*Note*: Abundant taxa are those above the threshold of 1% of total reads per sample and rare taxa are those below this threshold. Taxa presenting a ‘broad’ distribution are those appearing in ≥75% of the samples, ‘intermediate’ those present in >10% and <75% of samples, and ‘narrow’ those appearing in ≤10% of the samples.

### Synchrony of bacterioplankton

**FIGURE 4:**
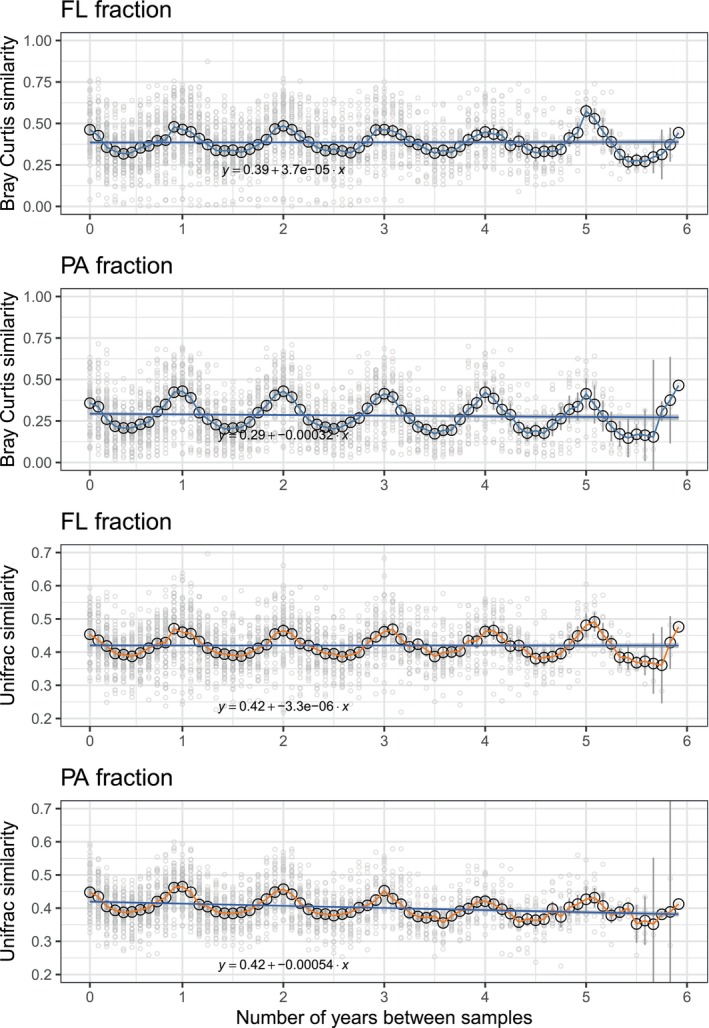
Bray‐Curtis and weighted UniFrac similarity between samples for each size fraction plotted against the time lag between each of them (time‐decay plot). Mean similarity values for each time lag are plotted in an empty black dot with standard error bars (background grey filled dots show each comparison). A linear regression is plotted, with 95% confidence intervals shown. A value of 1 indicates total similarity and a value of 0 indicates total dissimilarity.

To estimate patterns of community turnover, we calculated dissimilarity across samples separated by different time lags using two different distance metrics, Bray‐Curtis and UniFrac. Bray‐Curtis considers the proportion of taxa shared between pairs and their relative abundance, while UniFrac incorporates phylogenetic information. Figure 4 shows the time‐decay curve for both indices and fractions. In all cases communities separated by 1 year were those most similar whereas the lowest similarity existed between samples lagged 6 months. Bray–Curtis similarity varied from 0.32 to 0.46 in FL communities (mean 0.38) and 0.19–0.42 in the PA fraction (mean 0.28). UniFrac distance values were 0.68–0.73 for FL (mean 0.7) and 0.70–0.79 for PA (mean 0.73). Community differentiation barely increased over long periods, as samples separated by 1 year were similar to those from samples separated by various years, with a slight decline with increasing time lags for the particle‐associated fraction (0.12% change within the intercept baseline for Bray‐Curtis).

### Seasonality of bacterioplankton communities

**FIGURE 5:**
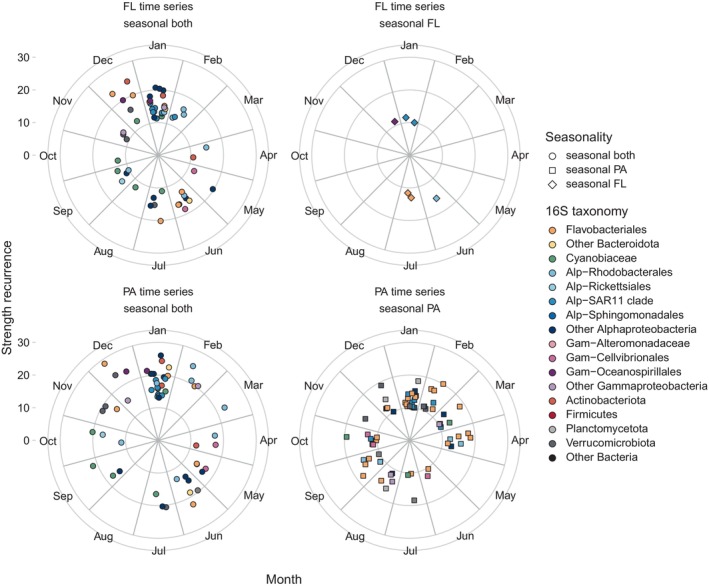
Polar plots representing the seasonal ASVs of the free‐living (FL) and particle‐associated (PA) communities. Different symbols indicate if the ASVs present seasonality in either one fraction or in both. ASVs are colour‐coded by taxonomic group. Higher strength recurrence values represent stronger seasonal signals.

The seasonal behaviour was explored for each ASV using Lomb Scargle time series analysis, a tool designed to detect periodic signals in unevenly spaced observations. From the 5187 ASVs in the dataset, only 815 ASVs were present in more than 10% of the samples and were thus considered in this analysis. Out of these 815, a total of 222 ASVs presented a significant periodicity, with 86 being seasonal in both fractions, 130 presenting seasonality only in the particle‐associated fraction and 6 being seasonal only in the free‐living fraction (Table 1). All periodicities found were annual. The seasonal ASVs represented 30% of the total relative abundance in the FL fraction and 46% of the reads in the PA fraction. Overall, ASVs that were seasonal in both fractions presented higher recurrence strength values indicating a stronger seasonal signal (Figure 5).

To further examine the relationship between seasonality and lifestyle preference, we computed the ‘particle‐association niche index’ (PAN index, Salazar et al., 2015) for each ASV (Figure S7). Briefly, this index summarizes the preference for one fraction over the other based on the occurrence and relative abundance of each ASV, taking an abundance‐weighted mean to account for the differences in sample number. This index allows positioning every ASV in a continuum describing its lifestyle preference with a value of 1 indicating exclusive presence in the PA fraction, 0 indicating exclusive presence in the FL fraction, and 0.5 indicating equal presence in both fractions. No clear correlation was found between the strength of the seasonal signal and the PAN index. Seasonal ASVs belonged to most of the taxonomic groups analysed. Figure S8 shows the relative abundance of ASVs showing either seasonal or non‐seasonal behaviour within each group. The general trend was that most groups contained only a small number of seasonal ASVs (average ~5% for FL and 7% in ~PA), yet these ASVs represented the most abundant ones (average across all groups ~30% of reads for FL and ~38% for PA). Nevertheless, there was an exception to this trend, the SAR11 clade in the particle‐associated fraction for which ~7% of its ASVs were seasonal but these only correspond to ~4% of the reads. For almost all taxonomic groups a noticeably higher count of seasonal ASVs existed in the PA as compared to the FL fraction (see Figure S8). Overall, Bacteroidetes and Gammaproteobacteria contained the highest number of seasonal ASVs. However, when taking into account the relative abundance, cyanobacterial and Rhodobacterales ASVs had higher values (Figure S8).

## DISCUSSION

Previous studies on microbial observatories in temperate zones have shown strong seasonal patterns of community structure and interannual recurrence of microbial taxa, but most of these studies did not distinguish between lifestyles. Here, we present the seasonal and interannual variability of free‐living (FL) and particle‐associated (PA) bacterial assemblies over 6 years in the Blanes Bay Microbial Observatory (BBMO). Our results demonstrate that both lifestyles present a marked seasonality and a high degree of recurrence. Up to now, we knew that in Blanes Bay free‐living bacterial assemblages, that represent most of the prokaryotic cell abundance in this site (Mestre, Borrull et al., 2017; Mestre, Ferrera et al., 2017), as well as eukaryotic pico‐ and nanoplankton communities present a recurrent pattern (Auladell et al., 2019, 2022; Giner et al., 2019). A short survey (2 years) also revealed gradual changes in prokaryotes inhabiting particles at this coastal site (Mestre et al., 2020) but whether the degree of recurrence in the particle‐associated communities was comparable to that of the free‐living microbes had not been tested.

The BBMO is an oligotrophic coastal ecosystem only occasionally impacted by terrestrial inputs (Guadayol et al., 2009). The annual cycle of temperature follows the typical of temperate regions, with chlorophyll and particulate primary production reaching their maxima in late winter‐early spring (Gasol et al., 2016). Although there is no data available from the period included in this work, a 3‐year study conducted at the same site showed that particulate organic carbon (POC) concentrations did not follow a consistent year‐to‐year pattern and that POC was decoupled from transparent exopolymer particles, which did show recurrent accumulation in early summer, and from particle mass, for which seasonal patterns are unclear due to the limited available data (only 1 year) (Ortega‐Retuerta et al., 2018). These results indicate that particle dynamics in Blanes Bay are complex likely due to the variability in the nature of particles. However, taking into account that most pelagic particles in this oligotrophic coastal site are likely biologically produced, as seen by the dynamics of TEPs, and that strong repeatable fluctuations in environmental conditions occur in these latitudes, we hypothesized that particle‐associated bacteria would also present a marked seasonality.

Bacterial communities showed recurring yearly patterns in alpha diversity regardless of their lifestyle. Previous studies in Blanes had observed a cyclic pattern in the diversity of free‐living bacteria as well as of eukaryotic pico‐ and nanoplankton communities (Auladell et al., 2022; Giner et al., 2019). This pattern had also been observed in other sites, such as the English Channel for bulk bacterial communities (Gilbert et al., 2012) or for free‐living bacteria, archaea, and autotrophic eukaryotes in the Bay of Banyuls (NW Mediterranean Sea) (Lambert et al., 2019). All these reports concur that the highest diversity values generally occur in autumn and winter but much less is known about the trends in particle‐associated communities. Mestre et al. (2020) reported increasing values of alpha diversity over 2 years from spring and summer towards autumn and early winter in all the 6 size fractions analysed, from 0.2 to 200 μm, even though there were fewer bacteria per unit of volume as the fraction increased. Our data shows that the long‐term pattern of particle‐associated communities is consistent with these observations, with maximal diversity values in the autumn‐winter transition. On the other hand, as a general rule, richness increases with increasing size fraction in several studies, explained by an increased number of ecological niches (Bižić‐Ionescu et al., 2015; Mestre, Borrull et al., 2017; Mestre, Ferrera et al., 2017; Rieck et al., 2015; Yeh & Fuhrman, 2022a; Yung et al., 2016), although higher diversity in free‐living populations have been exceptionally reported in surface (Roth Rosenberg et al., 2021; Trano et al., 2022) and also in deep marine waters (Salazar et al., 2015). Alpha diversity values in the PA communities in our study were higher than those from the FL fraction over all seasons. Interestingly, differences in diversity between fractions were lower in summer than in other seasons (Figure 1), which could reflect that there were fewer particles in surface waters during this season due to a lack of algal blooms or reduced resuspension, although these differences were not statistically significant (*p* > 0.05). The number of abundant ASVs was similar in the two fractions, while the PA contained many more rare ASVs, contributing thus to the observed higher richness values and supporting the idea that particles play an important role as hotspots for bacterial diversity, as previously proposed (Grossart, 2010).

In terms of community comparisons, samples were found to cluster based on season, making fraction a less informative factor to explain community similarity. Seasonal oscillations in the configuration of bacterial communities in particles have been reported before (Mestre et al., 2020). Community assembly in the two size fractions of our study was seasonal and recurrent for both the abundant and rare components. The temporal dynamics of the rare biosphere have barely been investigated and only for those rare components with a free‐living lifestyle (Alonso‐Sáez et al., 2015). The long ‘tail’ of rare species that microbial communities have (the so‐called ‘rare biosphere’) represents a persistent microbial seed bank that is crucial for maintaining resiliency in the face of environmental fluctuation (Jones & Lennon, 2010; Lynch & Neufeld, 2015; Pedrós‐Alió, 2012). Defining the seasonal variations of the rare members is necessary to fully understand the dynamics of microbial communities and their potential to respond to a changing environment and to sustain biogeochemical functions. In our study, a recurrent pattern of community assembly was observed for the abundant and rare members in the two fractions, although the strength of seasonality in explaining the observed patterns was higher for the abundant taxa.

Community composition was overall similar between fractions in terms of broad groups but some taxa were more abundant in either the free‐living or the particle‐associated fraction. The most evident cases were the SAR11 clade, whose abundance was lower in the PA assemblies, and the Verrucomicrobia that showed the opposite trend. These findings are consistent with previous studies (Freitas et al., 2012; Ganesh et al., 2014; López‐Pérez et al., 2016; Mestre, Borrull et al., 2017; Mestre, Ferrera et al., 2017; Yeh & Fuhrman, 2022a, 2022b). Members of the SAR11 clade are known to be abundant in the free‐living fraction of bacterioplankton and to have genome properties that allow them to be prevalent under oligotrophic conditions (Grote et al., 2012). On the contrary, Verrucomicrobia are highly specialized degraders of algal complex polysaccharides (Sichert et al., 2020), which could explain their preference for particles. Nevertheless, no clear trends were observed between taxonomy or lifestyle and seasonality. Lifestyle seems to be a phylogenetically conserved trait (Salazar et al., 2015) but we found no correlation between the PAN index, an indicator of lifestyle preference, and seasonality. Seasonal and non‐seasonal taxa were found in both fractions and similarly across most broad taxa. Our results thus indicate that seasonality does not broadly depend on phylogeny or lifestyle.

Further evidence on the comparable seasonality of free‐living and particle‐associated bacteria was provided by the clear annual recurrence patterns observed in both fractions, which is consistent with previous findings on the surface waters of the San‐Pedro Ocean Time series (SPOT) (Yeh & Fuhrman, 2022a). Time‐decay curves showed a high degree of stability in community turnover, indicating little year‐to‐year variation in bacterial assemblages in both fractions. However, the degree of predictability was slightly higher for the free‐living assemblages than for the particle‐associated ones only when comparing Bray–Curtis similarity vs. weighted UniFrac measure. The Bray‐Curtis metric does not consider phylogenetic relationships between ASVs and is sensitive to differences in abundance between taxa, weighting abundant taxa more than rare ones. On the other hand, weighted UniFrac accounts for taxa abundance and phylogenetic relatedness. The differences observed in the comparison between these two metrics suggest that the larger changes observed over time in the particle‐associated fraction respond to yearly variations in the abundance of the same taxa or to substitutions of closely related taxa, rather than to the establishment of different organisms over time. Community stability can be measured in terms of composition or function, and the degree to which functional and compositional stability are related may be variable (Shade et al., 2012). Besides, microbial communities harbour a certain degree of functional redundancy, meaning that changes in community composition may not necessarily lead to changes in function. However, some microbial functions are carried out only by a few taxa and there is a certain ecological coherence of closely related taxa (Auladell et al., 2022; Philippot et al., 2010; Tromas et al., 2018). Thus, the substitution of closely related taxa may have less impact on the functional stability of microbial communities than substitutions by distantly related taxa. Recently, Auladell et al. (2022) using data from the Blanes Bay Microbial Observatory, explored how conserved the seasonal niche preference is at different phylogenetic levels, from closely related taxa to broader taxonomic levels in the same time series, concluding that seasonal trends do not exist at the class level, while at the order and family ranks it depends on the patterns observed at the genus level. Additionally, for certain genera, niche similarity decreased as nucleotide divergence increased, which is a pattern compatible with the selection of similar taxa through environmental filtering. Accounting for phylogenetic relatedness may thus be crucial for better predicting the temporal dynamics of marine microbial communities.

Various studies have suggested that the seasonality of bacteria with different lifestyles is governed by different environmental variables (Mestre et al., 2020; Roth Rosenberg et al., 2021; Yung et al., 2016). However, we found that a similar suite of environmental factors were explanatory variables for both free‐living and particle‐associated bacteria and that the percentage of variability explained was comparable. This is in line with a recent report that compared FL and PA communities for over 14 years at the San‐Pedro Ocean Time series (SPOT) (Yeh & Fuhrman, 2022b). Yet in our study, like in many others, a significant part of the observed variability could not be explained by the commonly measured parameters (i.e., temperature, day length, chlorophyll, nutrients, etc.) suggesting that other factors and/or biotic interactions could be determinants of community structure. There is a growing awareness that taxa‐specific interactions may play critical roles in controlling plankton diversity and dynamics at both spatial and temporal scales (i.e., Lima‐Mendez et al., 2015; Needham & Fuhrman, 2016). In that sense, Yeh and Fuhrman (2022b) found that the structure of the phytoplankton community was a good predictor of the seasonality of particle‐associated prokaryotes while viral communities were so for the free‐living ones at SPOT. Another study unveiled higher bacteria‐phytoplankton associations in the particle‐associated communities than in the free‐living ones in the Southern Bay of Biscay (Spain) (Arandia‐Gorostidi et al., 2022). The dominant role of biotic interactions over environmental factors and taxa associations in Blanes Bay was recently revealed through network analyses of bacteria and protists (Krabberød et al., 2022). This study compared subnetworks in the picoplankton and nanoplankton fractions (corresponding respectively to the FL and PA fractions in our study) and found that nanoplankton networks were generally more connected than the picoplankton counterparts and bacteria‐eukaryotic interactions represented slightly higher percentages of the total interactions in the nanoplankton than in the picoplankton subnetworks. Among these, several associations were found between Alphaproteobacteria and phytoplankton groups, such as dinoflagellates, diatoms, cryptophytes, and Mamiellophyceae. Congruently, the seasonal dynamics of both free‐living and particle‐associated bacteria were partly explained in our study by the seasonal variation of protists, with the particle‐associated fraction having a higher mantel correlation between bacteria and protist community composition matrices. Interestingly, the variation of particle‐associated bacteria was better explained by correlations with phytoplankton data from the whole plankton size spectrum than that of free‐living communities. Marine bacteria depend to a large extent on substrates provided by phytoplankton and given the results observed here and in other reports (Arandia‐Gorostidi et al., 2022; Yeh & Fuhrman, 2022b), this dependence may be stronger for particle‐associated bacteria. Our results also support the idea that in this oligotrophic coastal site most small pelagic particles are of biological origin and most particle‐associated bacteria likely live in close association with phytoplankton, either through colonizing the phycosphere and establishing specific interactions (Seymour et al., 2017) or living on phytoplankton detritus. Whether viruses are significant determinants of the temporal dynamics of bacteria in this site, and if they do so at the same level for each fraction, remains unknown.

In summary, our study shows that both free‐living and particle‐associated bacterial communities persist over time, indicating that this is a fundamental property of microbial communities regardless of their lifestyle. The seasonal dynamics of these communities are shaped by pronounced and consistent changes in environmental conditions, alongside biotic interactions, which may exert a more significant impact on the variability of particle‐associated bacteria. Although both fractions display marked seasonality, their patterns diverge, underscoring the importance of monitoring the seasonal reappearance of different microbial community components to improve forecasts of their temporal dynamics in forthcoming scenarios. Future research focusing on analysing the seasonality of functional genes across various size fractions via metagenomics could provide insights into the contributions of bacteria with diverse lifestyles to key biogeochemical processes.

## AUTHOR CONTRIBUTIONS

**Isabel Ferrera:** Conceptualization; methodology; data curation; investigation; validation; supervision; funding acquisition; writing – original draft; writing – review and editing; visualization. **Adrià Auladell:** Methodology; data curation; formal analysis; writing – review and editing; investigation; visualization. **Vanessa Balagué:** Methodology; writing – review and editing. **Albert Reñé:** Methodology; writing – review and editing. **Esther Garcés:** Funding acquisition; writing – review and editing; resources. **Ramon Massana:** Methodology; funding acquisition; writing – review and editing; resources. **Josep M. Gasol:** Conceptualization; supervision; funding acquisition; writing – review and editing; resources.

## CONFLICT OF INTEREST STATEMENT

The authors declare no conflicts of interest.

## Supporting information


**Data S1.** Supporting Information.

## Data Availability

The data that support the findings of this study are openly available in the NCBI repository at https://www.ncbi.nlm.nih.gov/bioproject/PRJEB38773. All code is available on GitHub: https://github.com/adriaaulaICM/bbmo_timeseries_fraction.
