# Supplementary material for: Seasonal and interannual variability of the free‐living and particle‐associated bacteria of a coastal microbiome
Source: Environ Microbiol Rep. 2024 Jul 31;16(4):e13299. doi: 10.1111/1758-2229.13299 (PMC11289420; doi:10.1111/1758-2229.13299)
Supplement: Supplementary file 1 — Data S1. Supporting Information. [file EMI4-16-e13299-s001.pdf]

Supplementary Materials

Seasonal and interannual variability of the free-living and particle-associated bacteria of a coastal microbiome

Isabel Ferrera, Adrià Auladell, Vanessa Balagué, Albert Reñé, Esther Garcés, Ramon Massana, and Josep M. Gasol

Contents:

Table S1. Mantel tests comparing each pair of community composition matrices.
Table S2. Proportion of the variance explained by biological components
Table S3. Relative abundance of the different taxa in the Blanes Bay Microbial Observatory

Fig. S1. Distribution of abiotic variables across the time series
Fig. S2. Distribution of biotic variables across the time series
Fig. S3. Non-metrical multidimensional plots
Fig. S4. Violin plots of Bray-Curtis similarities between fractions
Fig. S5. Heatmap of the monthly mean relative contribution of main taxa
Fig. S6. ASVs in the FL against the PA fraction
Fig. S7. PAN Index
Fig. S8. Seasonal and non-seasonal ASVs

Supplementary Tables

Table S1. Results of the Mantel tests comparing each pair of community composition matrices. n: number of samples in the comparison.

	**Free-living bacteria** (16S rRNA)			**Particle-associated bacteria** (16S rRNA)		
	**R**	**p value**	**n**	**R**	**p value**	**N**
Phytoplankton community (chemtax)	0.090	0.024	57	0.264	0.001	54
Picoeukaryotes (18S rRNA)	0.595	0.001	69	0.580	0.001	69
Nanoeukaryotes (18S rRNA)	0.357	0.001	72	0.555	0.001	67

Table S2. Proportion of the variance of free-living and particle-associated bacterial communities explained by other biological components (pCCA constrained) after partitioning out the environmental effect (i.e., temperature and chlorophyll *a,* pCCA conditional). Signif: significance; n: number of samples in the comparison.

	**Free-living bacteria** (16S rRNA)				**Particle-associated bacteria** (16S rRNA)			
	**pCCA (constr.)**	**pCCA (condic.)**	**Signif.**	**n**	**pCCA (constr.)**	**pCCA (condic.)**	**Signif.**	**N**
Phytoplankton community (chemtax)	0.133	0.100	0.475	57	0.183	0.146	0.001	54
Picoeukaryotes (18S rRNA)	0.29	0.07	0.001	69	0.25	0.13	0.001	69
Nanoeukaryotes (18S rRNA)	0.05	0.07	0.05	72	0.07	0.13	0.001	67

Table S3. Relative abundance of the different taxa in the Blanes Bay Microbial Observatory across de 6-year period analyzed. Minimum (min), median, maximum (max) and standard deviation (sd) are shown for the free-living (FL) and particleassociated (PA) fractions.

	Free-living fraction				Particle-associated fraction			
**Taxonomic group**	**min**	**median**	**max**	**sd**	**min**	**median**	**max**	**sd**
Bacteroidetes								
Flavobacteriales	0.6	5.0	26.4	5.7	1.6	6.7	37.9	6.0
Other Bacteroidota	0.0	0.3	3.2	0.6	0.0	0.4	5.8	0.9
Cyanobiaceae	0.0	10.4	52.2	14.4	0.4	23.0	74.6	18.8
Alphaproteobacteria								
Rhodobacterales	0.2	5.7	54.6	9.5	1.0	4.6	32.3	7.3
Rickettsiales	0.0	0.8	2.9	0.7	0.0	0.5	2.5	0.5
SAR11 clade	0.1	43.3	78.4	15.0	0.6	24.9	56.8	14.3
Sphingomonadales	0.0	0.3	14.9	1.9	0.0	0.6	22.3	2.9
Other Alphaproteobacteria	0.5	9.2	37.8	4.9	1.3	6.5	21.9	3.6
Gammaproteobacteria								
Alteromonadales	0.0	0.7	47.2	8.3	0.0	0.9	24.7	4.0
Cellvibrionales	0.0	2.3	9.7	2.5	0.0	1.8	25.7	4.2
Oceanospirillales	0.0	1.1	9.8	1.7	0.1	1.3	42.0	5.3
Other Gammaproteobacteria	0.0	1.0	93.4	12.6	0.1	2.8	16.3	3.5
Actinobacteria	0.0	0.8	3.4	0.9	0.0	0.6	18.7	2.3
Firmicutes	0.0	0.0	1.7	0.3	0.0	0.3	16.9	2.3
Planctomycetes	0.0	0.0	0.2	0.0	0.0	0.5	10.5	1.7
Verrucomicrobia	0.0	1.2	34.4	4.3	0.1	3.2	42.6	7.5
Other Bacteria	0.0	0.1	8.1	1.1	0.0	0.4	6.0	1.0

Supplementary Figures

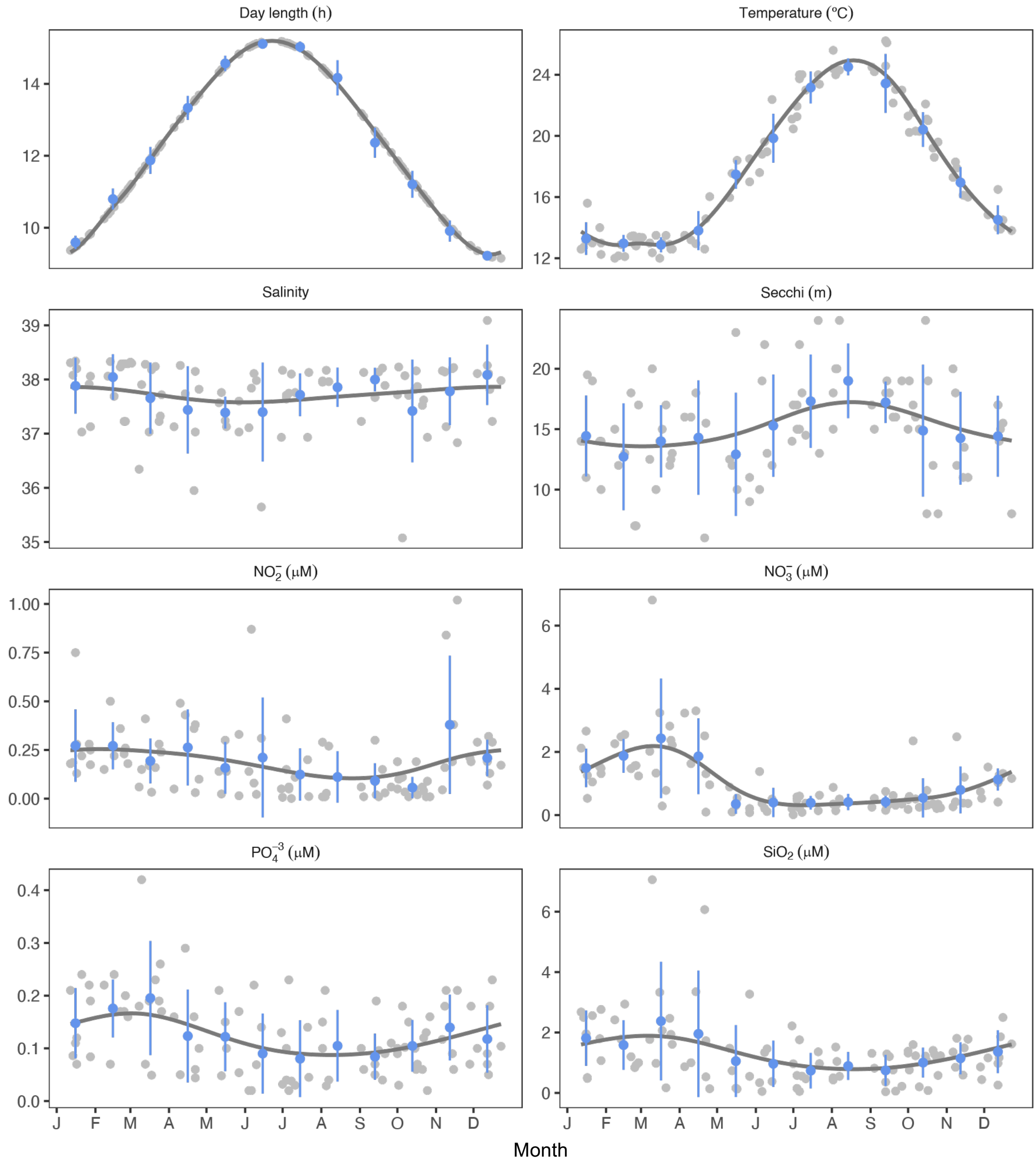

Supplementary Figure S1. Distribution of abiotic variables across the 6-year time-series. The Y-axis corresponds to the parameter value (units indicated in the plot title) and the X-axis corresponds to the day of the year (month is shown for orientation, with the axis ticks at the first day of each month). A generalized additive model is fitted to the data.

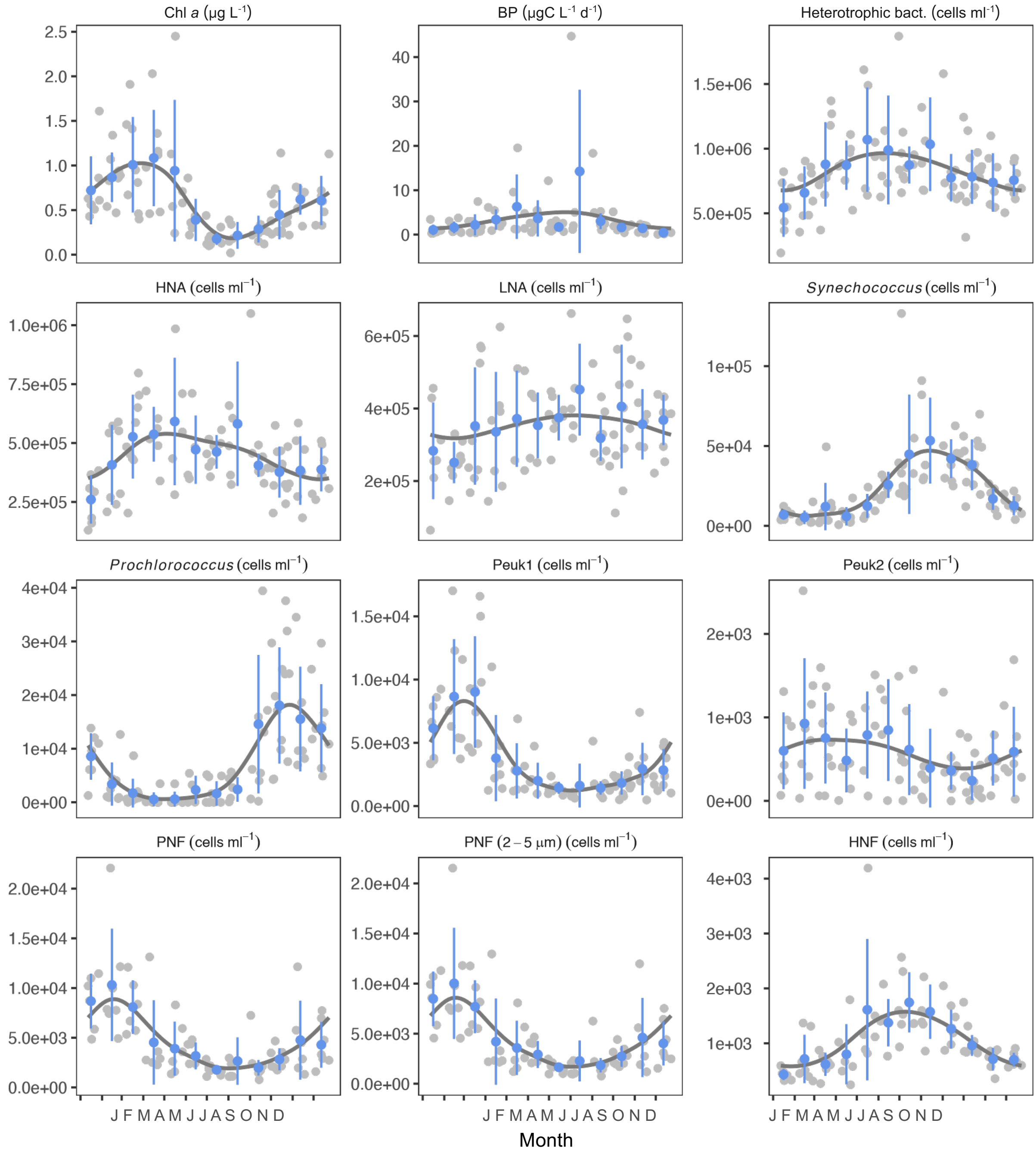

Supplementary Figure S2.Distribution of the biotic variables across the 6-year time-series. The Y-axis corresponds to the parameter value (units indicated in the plot title) and the X-axis corresponds to the day of the year (month is shown for orientation, with the axis ticks at the first day of each month). A generalized additive model is fitted to the data. Chl *a*: Chlorophyll *a*; BP: bacterial production; HNA: high nucleic acid content bacteria determined by flow cytometry; LNA: low nucleic acid content bacteria determined by flow cytometry; Peuk1: a cytometric population of picoeukaryotes (group I); Peuk2: a cytometric population of picoeukaryotes (group II); PNF: abundance of phototrophic nanoflagellates counted by epifluorescence microscopy; PNF(2-5 µm): PNF in the 2-5 µm size range; HNF: heterotrophic nanoflagellates counted by epifluorescence microscopy.

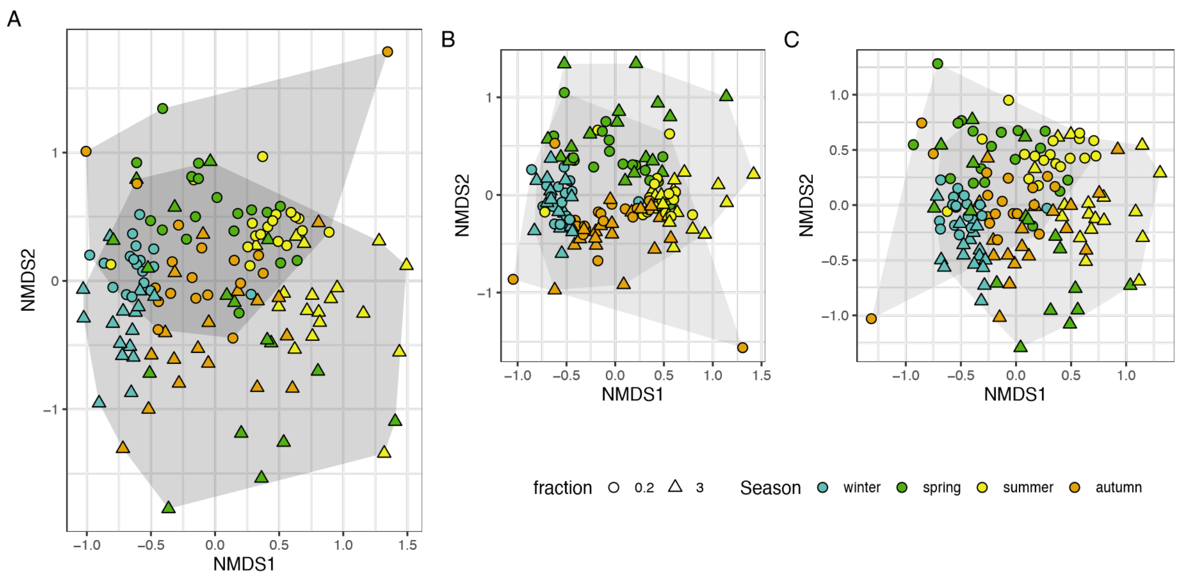

Supplementary Figure 3. Non-metrical multidimensional (nMDS) plots based on the ASV distributions of the entire dataset (A), the abundant ASVs (B), and the rare ASVs (C). Distance is derived from the Bray-Curtis dissimilarity calculated from the square root transformed relative abundance of each ASV. Free-living (FL) and particle-associated (PA) samples are indicated by different symbols, and seasons are color-coded. The grey background area delimits all samples from the same fraction.

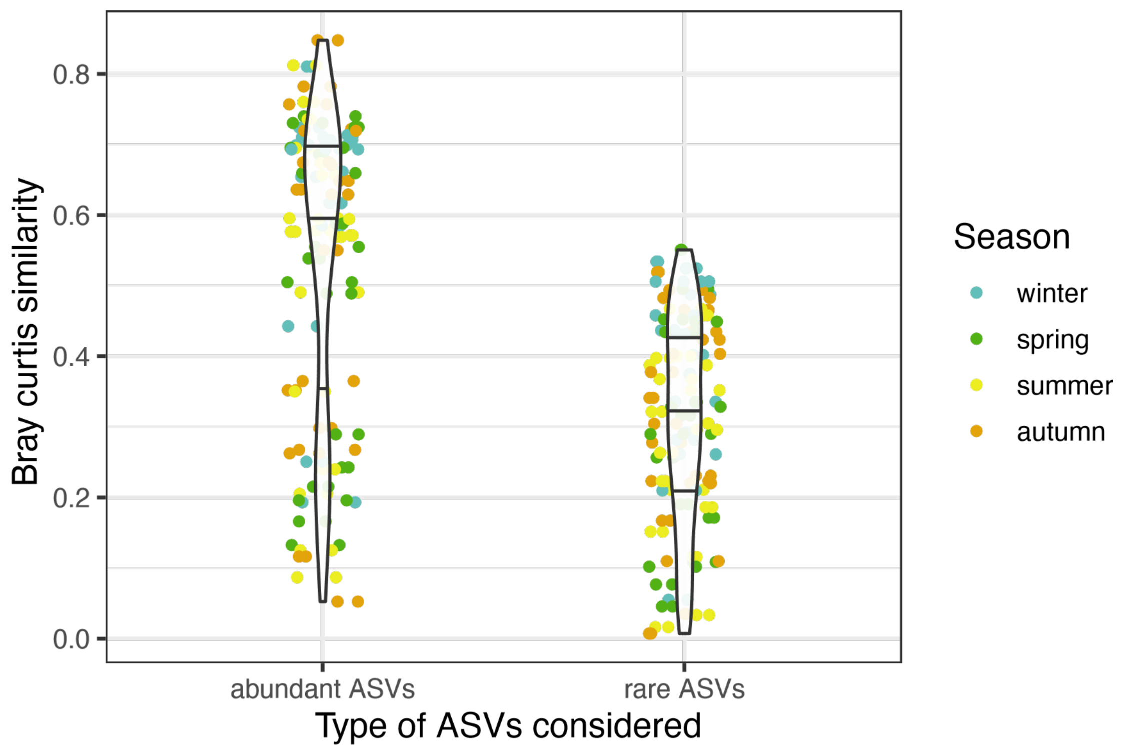

Supplementary Figure 4. Violin plots showing the Bray-Curtis similarity between the FL and PA fractions of each sample, plotted separately for the abundant (>1%) and rare (<1%) taxa. Each violin presents the median and the 25 and 75% quantiles. A value of 1 indicates total similarity and a value of 0 indicates total dissimilarity.

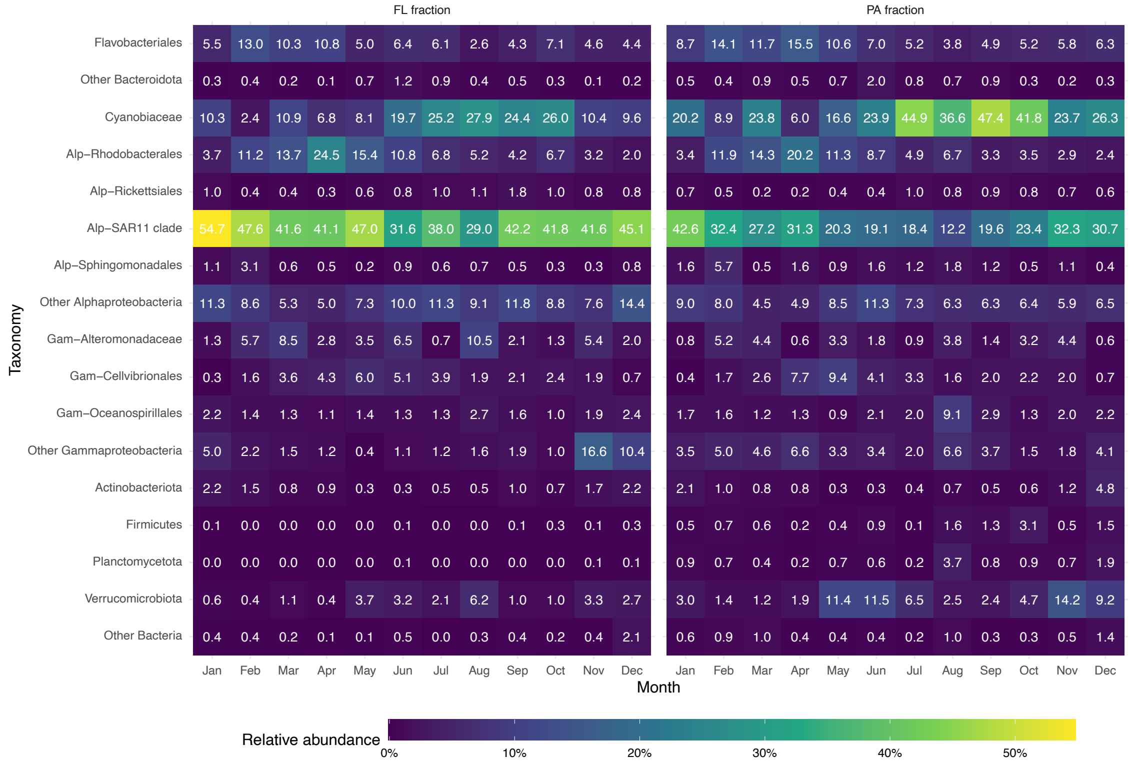

Supplementary Figure 5. Heatmap of the monthly mean relative contribution of main taxonomic groups in each fraction across the 6-year time series. FL: free-living, PA: particleassociated.

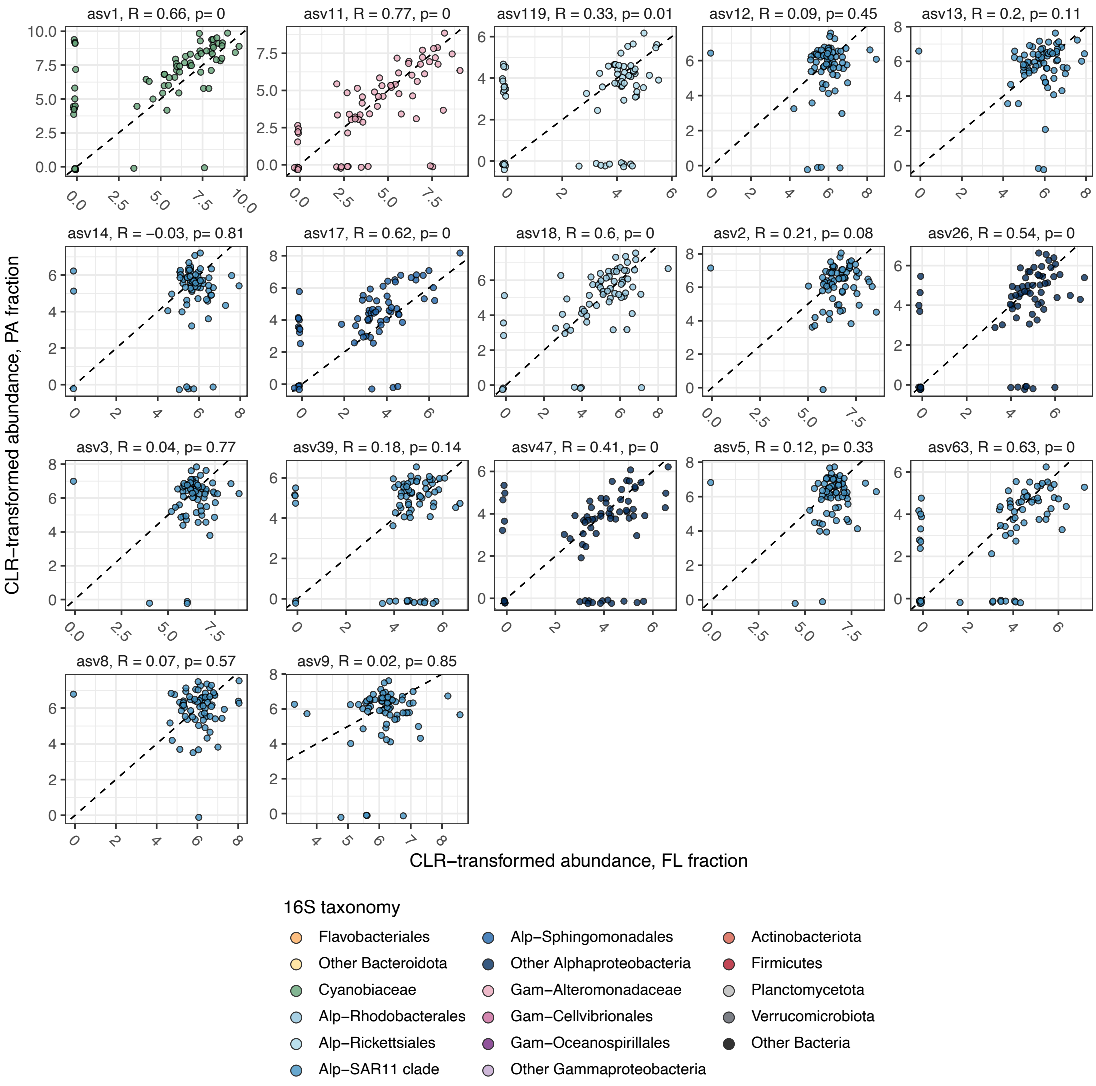

Supplementary Figure 6. Relative abundance of ASVs in the FL fraction (x axis) against the PA fraction (y axis). The ASVs in the subset were selected based on their occurrence (>75% samples) and their relative abundance (>1% relative abundance in at least one sample). ASVs are color coded based on their taxonomic assignment. Spearman correlation is showed in each panel.

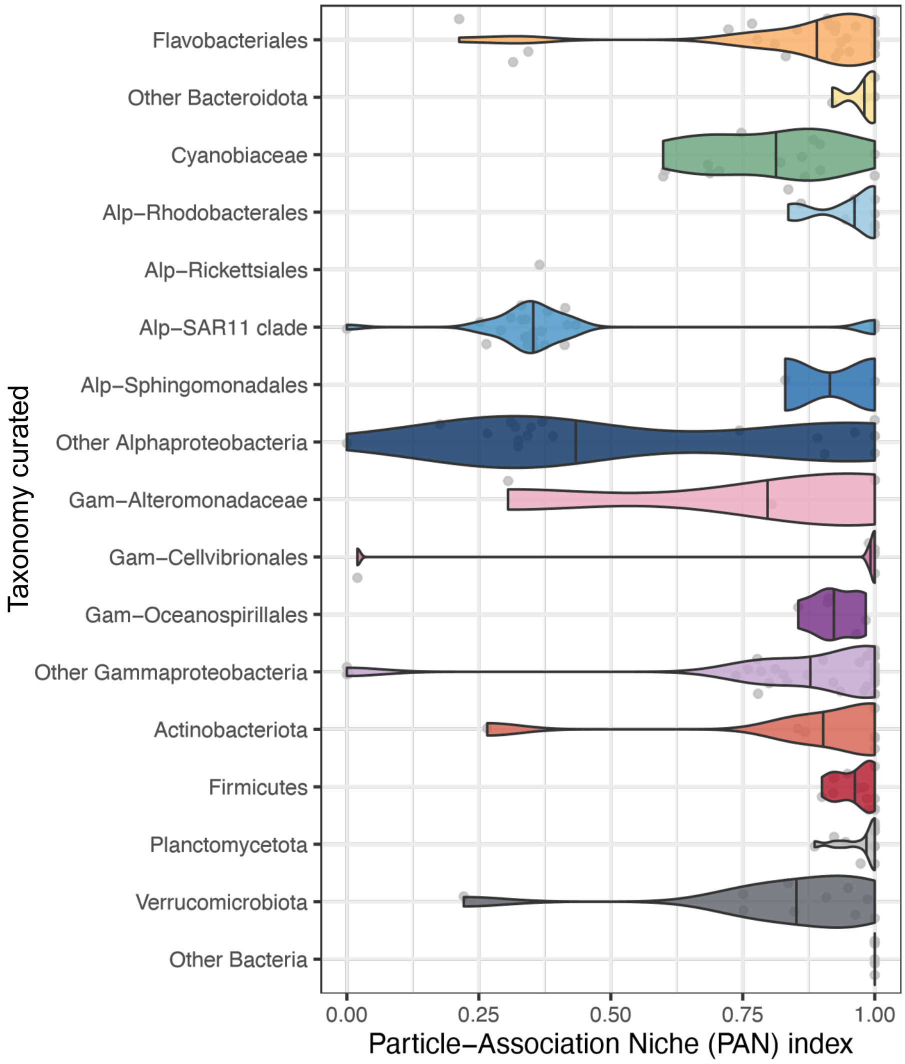

Supplementary Figure S7. Violin plots of the particle-association niche index (PAN index) for the main for the main taxa.

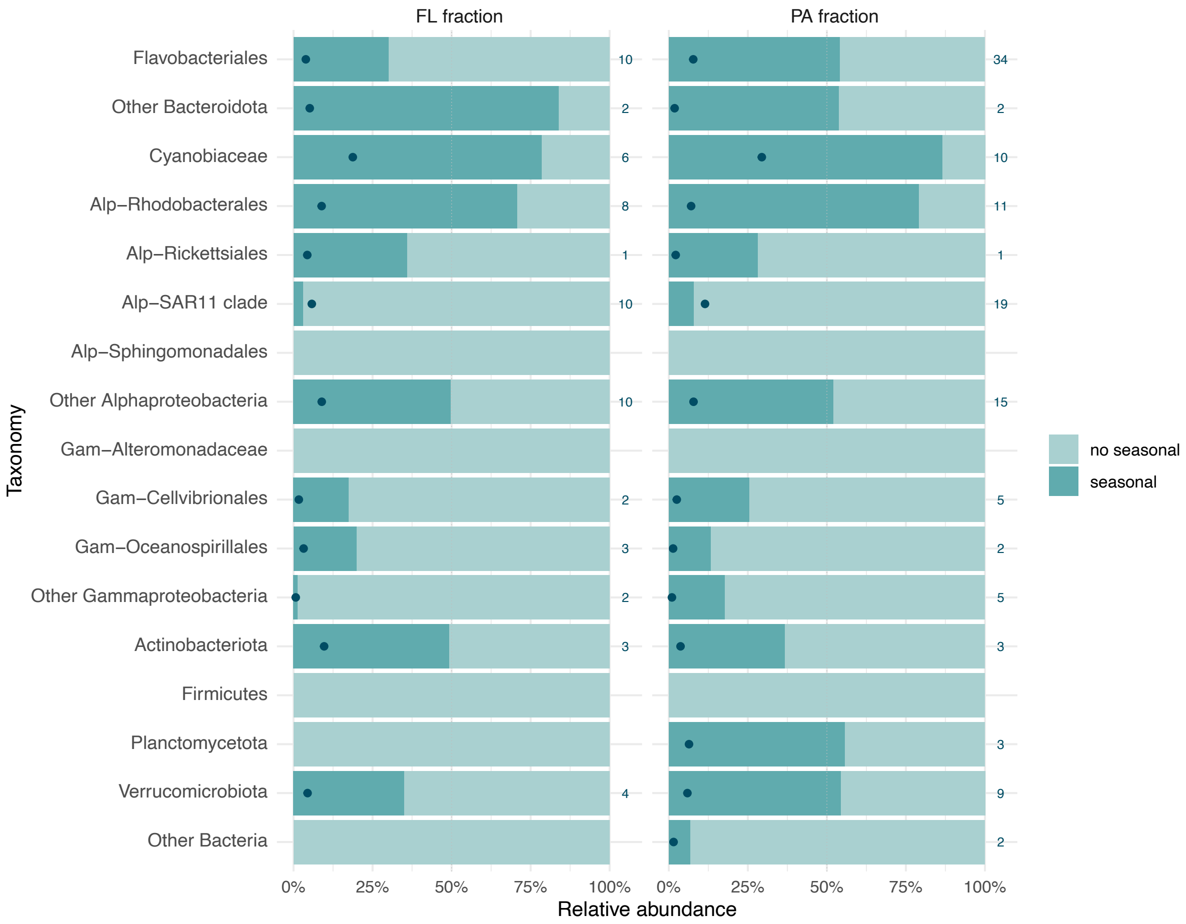

Supplementary Figure S8. Stacked bars showing the relative abundance of seasonal and non-seasonal ASVs. Dots represent the percentage of ASVs showing seasonality within each taxonomic group, and the number of ASVs is indicated on the right side of each panel.
